# NudC-like protein 2 restrains centriole amplification by stabilizing HERC2

**DOI:** 10.1038/s41419-019-1843-3

**Published:** 2019-08-19

**Authors:** Min Li, Xiaoyang Xu, Jun Zhang, Min Liu, Wei Wang, Ya Gao, Qiang Sun, Jiayu Zhang, Yunkun Lu, Fangwei Wang, Wei Liu, Tianhua Zhou, Yuehong Yang

**Affiliations:** 1 Department of Cell Biology and the Cancer Center of the Second Affiliated Hospital, Zhejiang University School of Medicine, Hangzhou, Zhejiang 310058 China; 2 Hangzhou Xiacheng Hospital of Integrated Traditional Chinese and Western Medicine, Hangzhou, Zhejiang 310004 China; 3 Life Sciences Institute and Innovation Center for Cell Signaling Network, Zhejiang University, Hangzhou, Zhejiang 310058 China; 4 Collaborative Innovation Center for Diagnosis and Treatment of Infectious Diseases, Hangzhou, Zhejiang 310003 China; 5 Department of Molecular Genetics, University of Toronto, Toronto, Canada

**Keywords:** Mitotic spindle, Organelles

## Abstract

Centriole duplication is tightly controlled to occur once per cell cycle, and disruption of this synchrony causes centriole amplification, which is frequently observed in many cancers. Our previous work showed that nuclear distribution gene C (NudC)-like protein 2 (NudCL2) localizes to centrosomes; however, little is known about the role of NudCL2 in the regulation of centrosome function. Here, we find that NudCL2 is required for accurate centriole duplication by stabilizing the E3 ligase HECT domain and RCC1-like domain-containing protein 2 (HERC2). Knockout (KO) of *NudCL2* using CRISPR/Cas9-based genome editing or depletion of NudCL2 using small interfering RNA causes significant centriole amplification. Overexpression of NudCL2 significantly suppresses hydroxyurea-induced centriole overduplication. Quantitative proteomic analysis reveals that HERC2 is downregulated in *NudCL2* KO cells. NudCL2 is shown to interact with and stabilize HERC2. Depletion of HERC2 leads to the similar defects to that in *NudCL2*-downregulated cells, and ectopic expression of HERC2 effectively rescues the centriole amplification caused by the loss of NudCL2, whereas the defects induced by HERC2 depletion cannot be reversed by exogenous expression of NudCL2. Either loss of NudCL2 or depletion of HERC2 leads to the accumulation of ubiquitin-specific peptidase 33 (USP33), a centrosomal protein that positively regulates centriole duplication. Moreover, knockdown of USP33 reverses centriole amplification in both *NudCL2* KO and HERC2-depleted cells. Taken together, our data suggest that NudCL2 plays an important role in maintaining the fidelity of centriole duplication by stabilizing HERC2 to control USP33 protein levels, providing a previously undescribed mechanism restraining centriole amplification.

## Introduction

Centrioles are cylindrical, microtubule-based structures that are essential for the formation of centrosomes, cilia, and flagella^1,2^. In general, a pair of centrioles recruits and organizes the pericentriolar material (PCM) to form a mature centrosome, which plays important roles in regulating cell shape, polarity, motility, mitosis, and cytokinesis^1,3^. In mammalian cells, centriole duplication occurs once per cell cycle, and is highly coordinated with DNA replication^4^. Centriole duplication consists of sequential steps that occur in a semiconservative and cell cycle-dependent manner. First, a pair of centrioles disengages during mitotic exit and early G1 phase. Then, a single new centriole, termed procentriole, begins to assemble adjacent to each pre-existing parental centriole in the G1/S transition, elongates through S and G2 phases, and finally becomes fully mature in the early phase of mitosis^4,5^. During this process, accurate control of the centriole number is crucial to maintain the number of centrosomes. Dysregulation of this process may cause centriole amplification, which leads to extranumerary centrosomes and results in genome instability^2,4^.

Nuclear distribution gene C (NudC)-like protein 2 (NudCL2) was cloned and characterized as a new homolog of NudC in mammalian cells^6^. In the filamentous fungus *Aspergillus nidulans*, NudC was first identified as an upstream regulator of NudF (a homolog of the human lissencephaly 1 gene product, LIS1) in the control of nuclear movement^7,8^. Mammalian NudC plays crucial roles in diverse cellular processes including cell division, neuronal migration, and ciliogenesis^9^. Our previous study has reported that NudCL2 plays an important role in regulating the LIS1/dynein pathway by enhancing the interaction between LIS1 and heat-shock protein 90 (Hsp90) to stabilize LIS1^6^. Additional data demonstrated that NudCL2 functions as an Hsp90 cochaperone to stabilize subunits of the ring-shaped cohesin complex that mediates sister chromatid cohesion^10^. Interestingly, NudCL2 has been shown to be associated with centrosomes in human cells^6^; however, little is known about the potential role of NudCL2 in centrosomes.

In this study, we provide evidence that NudCL2 is crucial for accurate centriole duplication. NudCL2 interacts with and stabilizes the E3 ligase HECT domain and RCC1-like domain-containing protein 2 (HERC2). Either downregulation of NudCL2 or depletion of HERC2 causes centriole amplification. Ectopic expression of HERC2 reverses the centriole defects induced by NudCL2 deletion, but not vice versa. Thus, these data suggest that NudCL2 suppresses centriole amplification by stabilizing HERC2.

## Results

### NudCL2 is associated with centrosomes

**Fig. 1:**
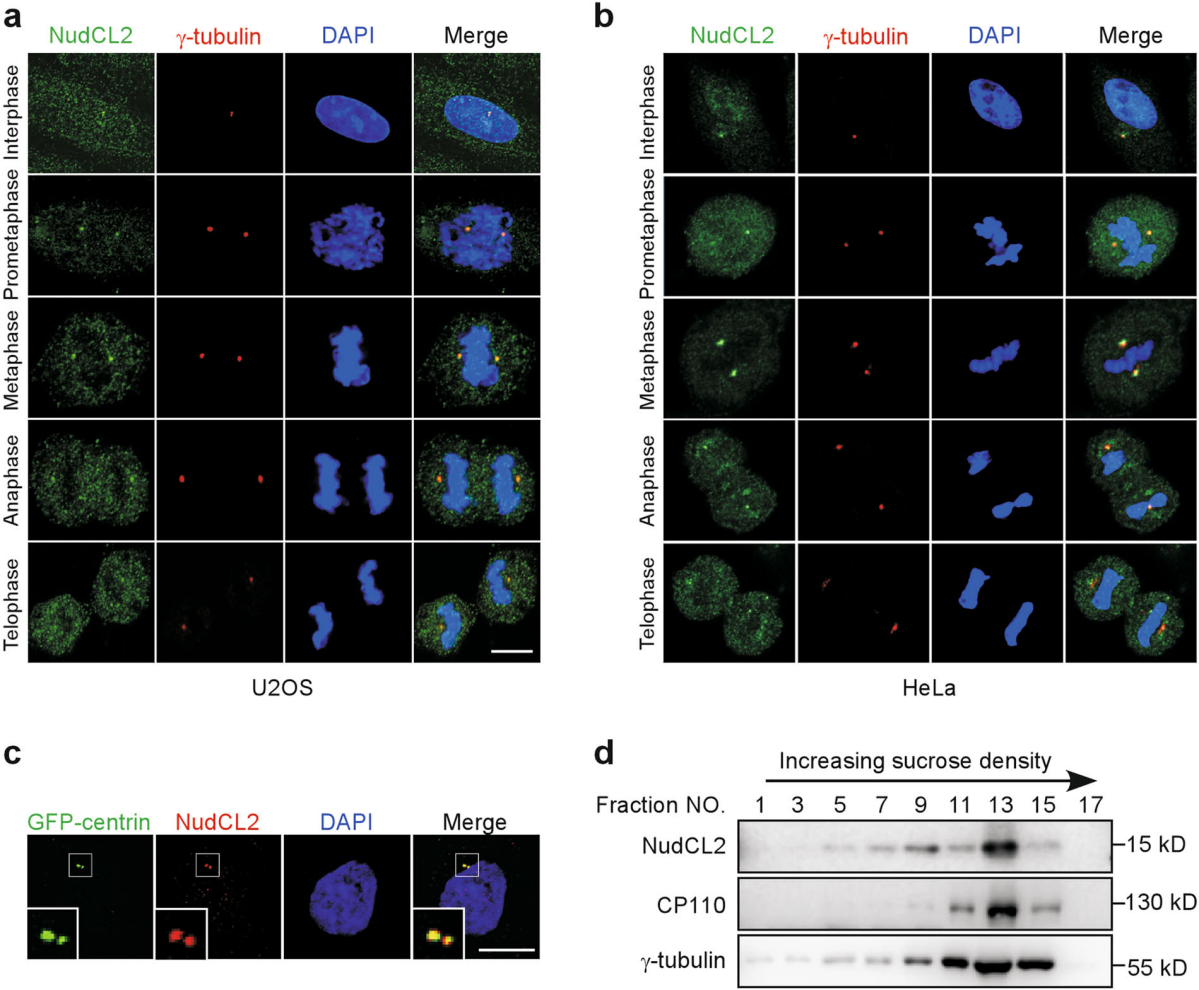
Nuclear distribution gene C (NudC)-like protein 2 (NudCL2) is localized to centrosomes. **a**, **b** U2OS and HeLa cells were fixed and subjected to immunofluorescence analyses with anti-NudCL2 (green) and anti-γ-tubulin (red) antibodies, respectively. **c** NudCL2 is colocalized with centrin. HeLa cells stably expressing green-fluorescent protein (GFP)-centrin were fixed and processed for immunofluorescence analysis with anti-NudCL2 (red) antibody. Higher magnifications of the boxed regions are displayed. **d** NudCL2 is cosedimented with centrosomes. U2OS cells were treated with 10 μg/ml nocodazole and 5 μg/ml cytochalasin B for 1.5 h. Lysates of the cells were fractionated by sucrose gradient centrifugation and applied for western blot analysis with the indicated antibodies. DNA was visualized with 4′,6-diamidino-2-phenylindole (DAPI) (blue). Scale bars, 10 μm

Our previous work indicated that NudCL2 is localized to centrosomes in HeLa cells^6^. To confirm the centrosome localization of NudCL2 during cell cycle progression, we performed immunofluorescence staining with anti-NudCL2 and anti-γ-tubulin (a centrosomal marker) antibodies in U2OS and HeLa cells and found that NudCL2 was colocalized with γ-tubulin throughout the cell cycle (Fig. 1a, b). Subsequent results revealed that NudCL2 was also associated with green-fluorescent protein (GFP)-centrin (a centriolar marker) that was stably expressed in HeLa cells (Fig. 1c). Moreover, sucrose density gradient centrifugation experiments showed that NudCL2 was cosedimented with γ-tubulin and centrosomal protein of 110 kDa (CP110) in U2OS cells (Fig. 1d). These data indicate that NudCL2 is a centrosome-associated protein.

### Downregulation of NudCL2 causes centriole amplification

**Fig. 2:**
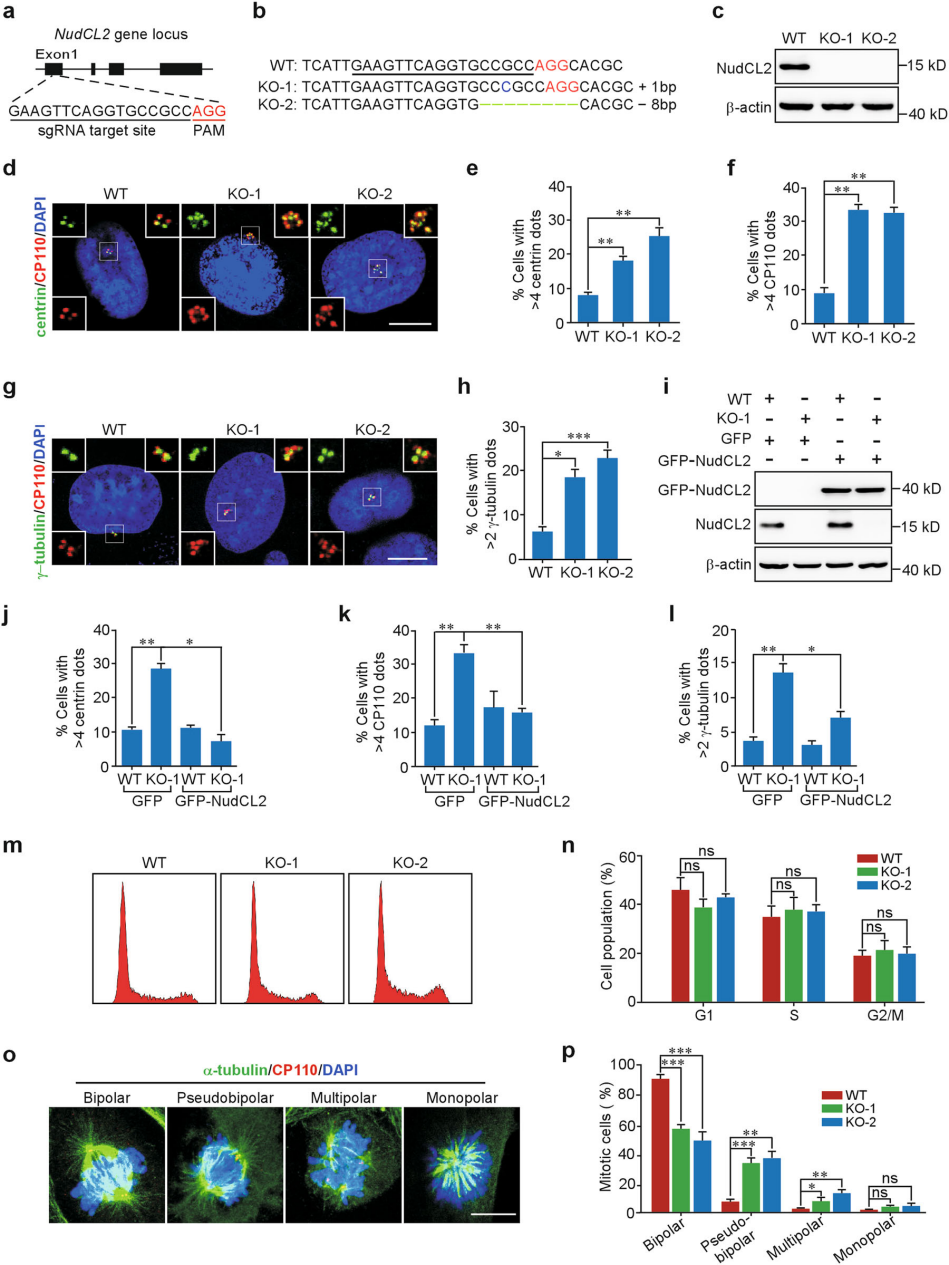
Downregulation of nuclear distribution gene C-like protein 2 (NudCL2) leads to centriole amplification. **a** Schematic representation of *NudCL2* gene targeting strategy. **b** Indel mutations of the *NudCL2* DNA locus in two *NudCL2* knockout cell lines. **c** Western blot analysis of NudCL2 protein in control and *NudCL2* KO U2OS cells. β-actin, a loading control. **d**–**f** Control and *NudCL2* KO U2OS cells were fixed and processed for immunofluorescence analysis with anti-centrin (green) and anti-CP110 (red) antibodies. Higher magnifications of the boxed regions are displayed. The frequencies of cells with more than four centrin and four CP110 dots were calculated, respectively. **g**, **h** Control and *NudCL2* KO U2OS cells were fixed and stained with anti-γ-tubulin (green) and anti-CP110 (red) antibodies. Higher magnifications of the boxed regions are shown. The number of cells with more than two γ-tubulin dots was plotted. **i**–**l** Control and *NudCL2* KO U2OS cells were transfected with green-fluorescent protein (GFP)-NudCL2 or GFP vector for 48 h and subjected to western blot and immunofluorescence analyses, respectively. β-actin, a loading control. The frequencies of cells with more than four centrin, four CP110, and two γ-tubulin dots were plotted, respectively. **m**, **n** The cell cycle distribution of control and *NudCL2* KO U2OS cells was analyzed by flow cytometry. **o**, **p** Cells were fixed and immunostained with anti-α-tubulin (green) and anti-CP110 (red) antibodies. Representative images of mitotic cells with bipolar, pseudobipolar, multipolar, or monopolar spindles are shown. The percentages of cells with various mitotic phenotypes were calculated. DNA was visualized with 4′,6-diamidino-2-phenylindole (DAPI) (blue). Scale bars, 10 μm. Quantitative data are expressed as the mean ± SD (at least three independent experiments). More than 150 cells were counted in each experiment. *p *<* 0.05, **p *<* 0.01, ***p < 0.001, ns, no significance, Student’s *t* test

To explore the role of NudCL2 in centrosome function, we employed CRISPR/Cas9-mediated genome editing to knock out *NudCL2* in mammalian cells. A CRISPR/Cas9 plasmid with a short guide RNA (sgRNA) that recognizes the first exon of *NudCL2* was constructed and transfected into U2OS cells (Fig. 2a). PCR amplification of genomic DNA followed by Sanger sequencing revealed indels that are predicted to cause frameshift mutations at the *NudCL2* DNA locus (Fig. 2b). Immunoblotting confirmed that NudCL2 protein disappeared in the mutant cells (Fig. 2c). In *NudCL2* knockout (KO) cells at interphase, the number of cells with more than four centrin, four CP110, or two γ-tubulin dots increased approximately three-fold compared to the wild-type (WT) cells (Fig. 2d–h), suggesting that loss of NudCL2 causes centriole amplification. The similar results were observed in *NudCL2* KO DLD1 cells and NudCL2-depleted CAL51 cells (Supplementary Figs. 1 and 2). Moreover, the increase in centriole number observed in *NudCL2* KO cells was significantly reversed by ectopic expression of NudCL2 (Fig. 2i–l). Given that cell cycle arrest may induce centriole amplification^2,11^, we determined whether centriole amplification induced by NudCL2 deletion resulted from a change in cell cycle progression in *NudCL2* KO cells. Fluorescence-activated cell sorting (FACS) analysis showed that there was no significant difference between the WT and *NudCL2* KO cells (Fig. 2m, n). Together, these data indicate that NudCL2 plays an important role in restraining centriole amplification.

Centrosomes are essential for bipolar spindle assembly and accurate chromosome segregation in mammalian cells^1^. Centriole amplification leads to supernumerary centrosomes in the subsequent cell cycle, which cluster to generate pseudobipolar spindles after transient spindle multipolarity, promoting chromosome missegregation^2,12–15^. To investigate the effects of NudCL2 deletion on mitotic spindle formation, we performed immunostaining analysis with anti-α-tubulin and anti-CP110 antibodies. The data showed that the frequency of cells exhibiting pseudobipolar spindles was significantly increased in *NudCL2* KO cells compared with that in WT cells (Fig. 2o, p), implying that loss of NudCL2 influences the formation of bipolar spindles.

### Overexpression of NudCL2 suppresses centriole overduplication

**Fig. 3:**
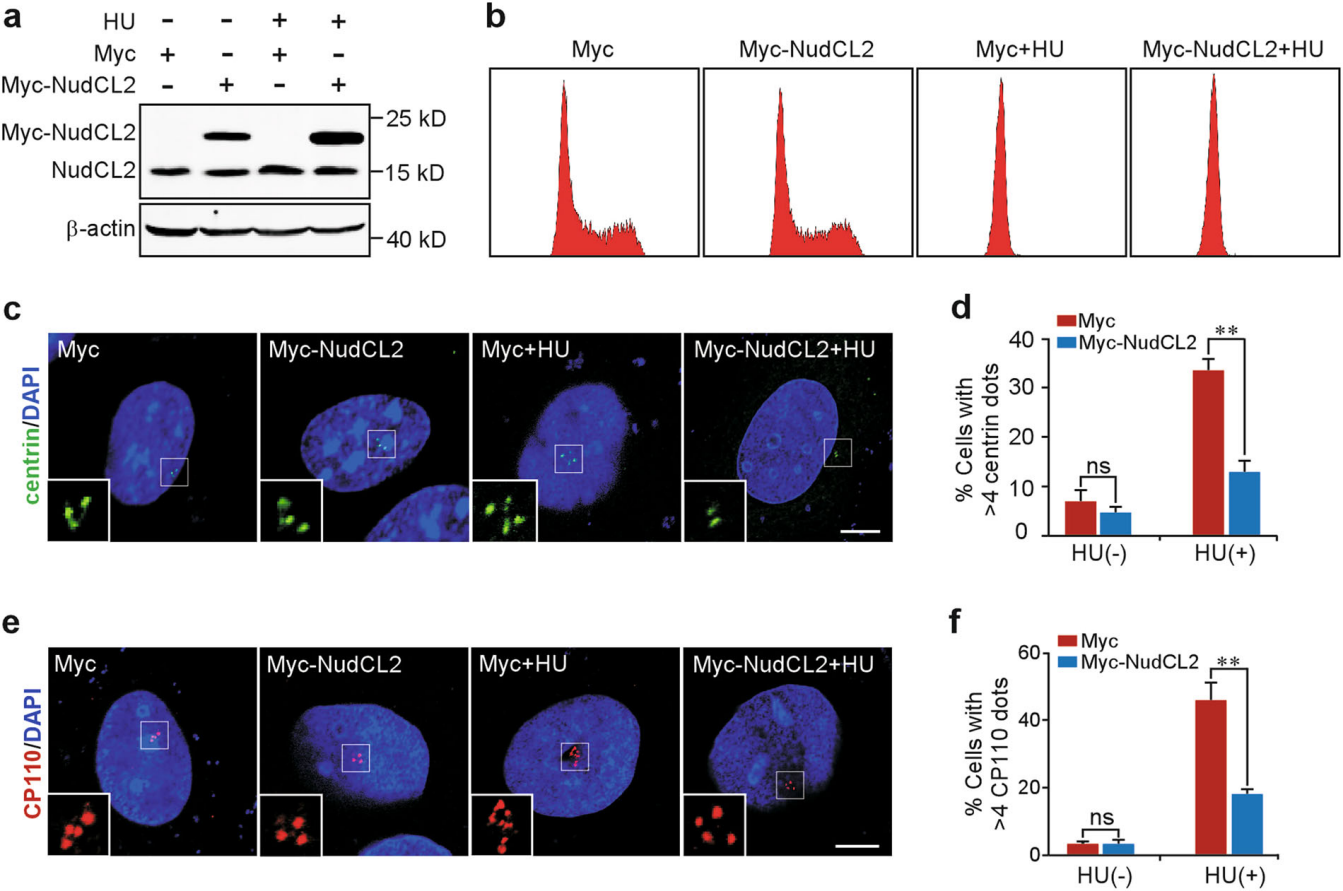
Overexpression of nuclear distribution gene C-like protein 2 (NudCL2) suppresses centriole overduplication. U2OS cells transfected with Myc-NudCL2 or Myc vector were treated with or without hydroxyurea (HU) for 48 h, and then subjected to the following analyses. **a** Western blot analysis of the expression of Myc-NudCL2. β-actin, a loading control. **b** Flow cytometry analysis of the cell cycle distribution. **c**–**f** Immunofluorescence analyses were carried out by using anti-centrin (green) or anti-CP110 (red) antibodies. Higher magnifications of the boxed regions are shown. The frequencies of cells with more than four centrin or four CP110 dots were plotted. DNA was visualized with 4′,6-diamidino-2-phenylindole (DAPI) (blue). Scale bars, 10 μm. Quantitative data are expressed as the mean ± SD (at least three independent experiments). More than 300 cells were counted in each experiment. **p < 0.01, ns, no significance, Student’s *t* test

Treatment with hydroxyurea (HU, a DNA synthesis inhibitor) uncouples centriole duplication from DNA replication and induces multiple rounds of centriole duplication in a prolonged S phase in U2OS cells, resulting in centriole amplification^16,17^. To confirm the role of NudCL2 in restraining centriole amplification, U2OS cells were transfected with Myc-NudCL2 followed by HU treatment. We found that NudCL2 overexpression did not obviously affect cell cycle progression as determined by FACS analysis (Fig. 3a, b). Immunostaining analyses with anti-centrin and anti-CP110 antibodies revealed that, upon HU treatment, the percentage of cells with more than four centrioles was significantly lower in Myc-NudCL2-expressing cells than in control cells (Fig. 3c–f), indicating that overexpression of NudCL2 suppresses centriole overduplication induced by HU treatment.

### Downregulation of NudCL2 induces HERC2 degradation

**Fig. 4:**
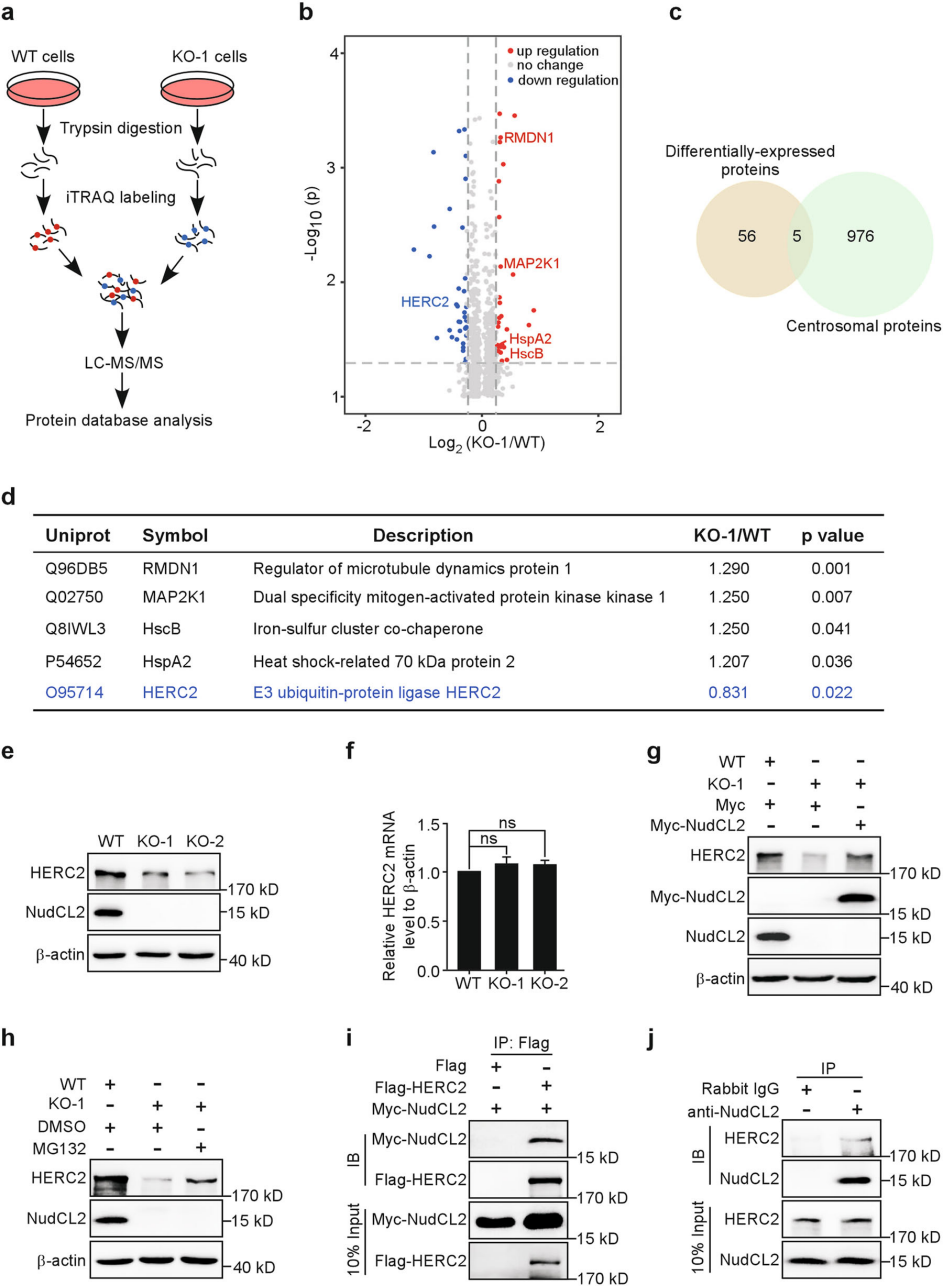
Downregulation of nuclear distribution gene C-like protein 2 (NudCL2) induces HECT domain and RCC1-like domain-containing protein 2 (HERC2) degradation. **a** Schematic representation of the isobaric tags for relative and absolute quantitation (iTRAQ)-based quantitative proteomic analysis. **b** Volcano plot showing *p* values (−log 10) versus the protein ratio of KO-1/WT cells (log 2). Proteins exhibiting a fold change >1.2 and *p* <0.05 were defined as “upregulation” (red dots), and those with a fold change <0.83 and *p* <0.05 were shown as “downregulation” (blue dots). Others were defined as “no change” (gray dots). **c** Venn diagram showing the overlap between the differentially expressed proteins from the quantitative proteomic analysis and the centrosomal proteins from the centrosome database. **d** Five centrosomal proteins that overlap in Fig. 4c are shown. **e** Western blot analysis of HERC2 protein in control and *NudCL2* KO U2OS cells using the indicated antibodies. β-Actin, a loading control. **f** Quantitative reverse transcription PCR (RT-PCR) analysis of *HERC2* messenger RNA (mRNA) in control and *NudCL2* KO U2OS cells. Quantitative data are expressed as the mean ± SD (at least three independent experiments). ns, no significance, Student’s *t* test. **g** Control and *NudCL2* KO U2OS cells were transfected with Myc-NudCL2 or Myc vector for 48 h and subjected to western blot analysis with anti-HERC2 and anti-NudCL2 antibodies. β-actin, a loading control. **h** Control and *NudCL2* KO U2OS cells were treated with MG132 or dimethyl sulfoxide (DMSO) for 2 h. Lysates of the cells were applied for western blot analysis with anti-HERC2 and anti-NudCL2 antibodies. β-actin, a loading control. **i** U2OS cells transfected with the indicated vectors for 48 h were harvested and lysed in TBSN (20 mM Tris [pH 8.0], 150 mM NaCl, 0.5% Nonidet P-40, 5 mM EGTA, 1.5 mM EDTA, 0.5 mM Na3VO4, 20 mM p-nitrophenyl phosphate) lysis buffer. Immunoprecipitation analysis was performed using the indicated antibodies. **j** HEK-293 cells were harvested and lysed. Immunoprecipitation analysis was carried out using the indicated antibodies

Previous work in our lab indicated that NudCL2 plays an important role in the regulation of protein stability^6,10^. To determine the mechanism of NudCL2 in centriole duplication, we performed isobaric tags for relative and absolute quantitation (iTRAQ)-based quantitative proteomic analysis using *NudCL2* KO cells, and found that 61 proteins were differentially expressed (KO-1/WT fold change >1.2 or <0.83, *p* < 0.05) (Fig. 4a, b). A range of centrosomal proteins have been reported to regulate centriole duplication^1^. To identify the potential centrosomal proteins that may be involved in the function of NudCL2 in suppressing centriole amplification, we compared our quantitative proteomics data with centrosome-associated proteins from centrosome database (http://centrosome.dacya.ucm.es)^18^. We found that five centrosomal proteins overlapped in the two datasets, including HERC2, heat-shock cognate B, heat-shock protein family A member 2, mitogen-activated protein kinase kinase 1, and regulator of microtubule dynamics 1 (Fig. 4c, d). Among these proteins, only HERC2 was downregulated in *NudCL2* KO cells, suggesting that HERC2 may be stabilized by NudCL2.

To confirm the downregulation of HERC2 induced by NudCL2 deletion, we performed western blot and reverse transcription PCR (RT-PCR) experiments. The results showed that the protein level of HERC2, but not its messenger RNA (mRNA) level, was substantially reduced in *NudCL2* KO U2OS cells compared to WT cells (Fig. 4e, f). In addition, KO of NudCL2 in DLD1 cells or depletion of NudCL2 in CAL51 cells also resulted in an obvious decrease in HERC2 protein levels (Supplementary Fig. 3). The reduction in HERC2 protein levels was rescued by ectopic expression of NudCL2 in *NudCL2* KO cells (Fig. 4g). In addition, cycloheximide (CHX) chase analysis revealed that the rate of HERC2 degradation was faster in *NudCL2* KO cells than in WT cells (Supplementary Fig. 4), implying that NudCL2 may be involved in the regulation of HERC2 protein stability. Furthermore, we employed the proteasome inhibitor MG132 to treat *NudCL2* KO cells and found that HERC2 degradation was decreased (Fig. 4h). Additional immunoprecipitation data showed that NudCL2 interacted with HERC2 (Fig. 4i, j). Together, these data strongly imply that NudCL2 plays an important role in stabilizing HERC2.

### HERC2 is involved in the regulation of centriole duplication in *NudCL2* KO cells

**Fig. 5:**
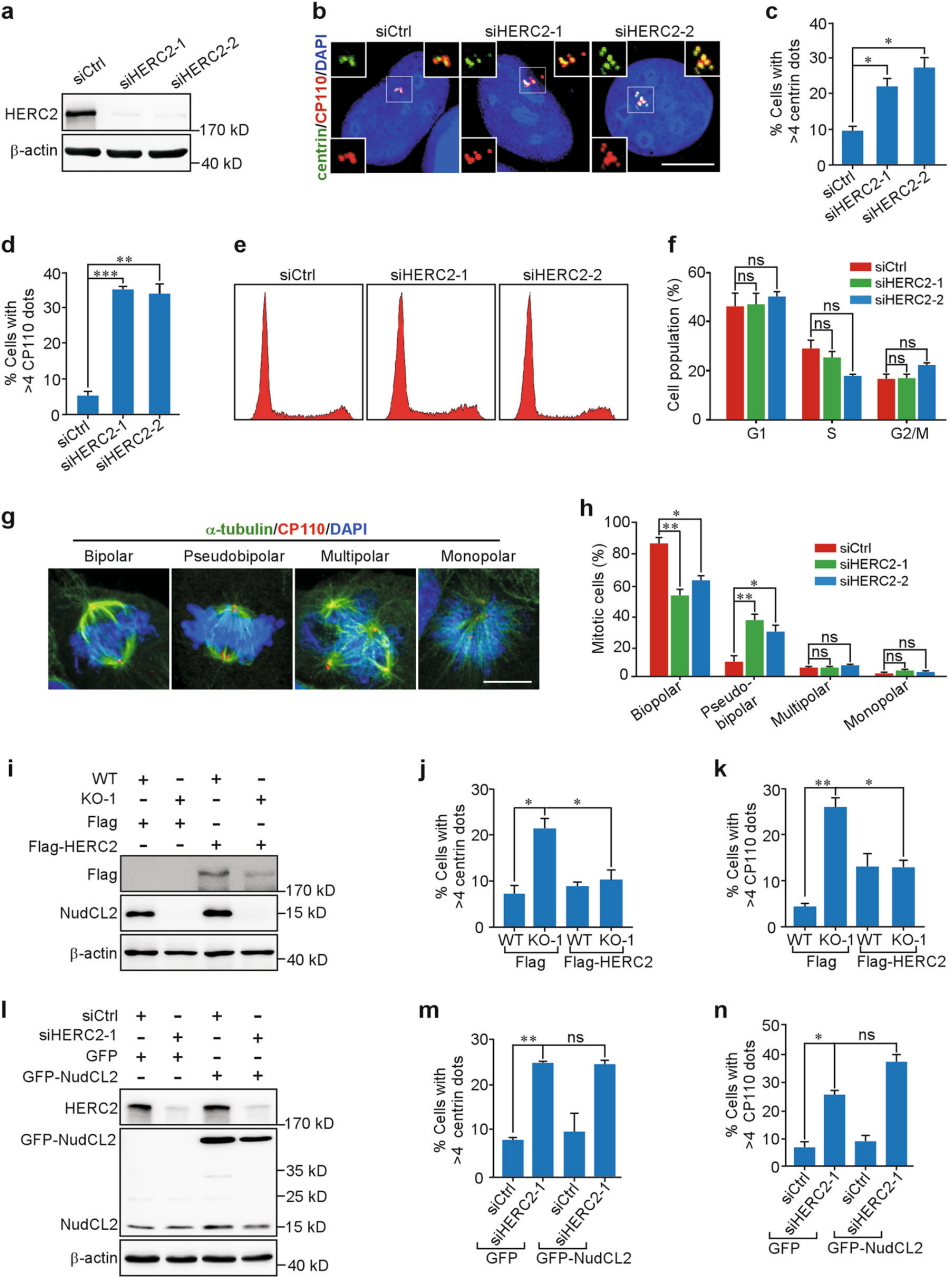
HECT domain and RCC1-like domain-containing protein 2 (HERC2) is involved in the regulation of centriole duplication in nuclear distribution gene C-like protein 2 (*NudCL2*) knockout cells. **a** U2OS cells were transfected with control or HERC2 small interfering RNAs (siRNAs) for 72 h and subjected to western blot analysis using anti-HERC2 antibody. β-actin, a loading control. **b**–**d** U2OS cells transfected with the indicated siRNAs were fixed and processed for immunofluorescence analysis with anti-centrin (green) and anti-CP110 (red) antibodies. Higher magnifications of the boxed regions are displayed. The frequencies of cells with more than four centrin and four CP110 dots were calculated, respectively. **e**, **f** Flow cytometry analysis of the cell cycle distribution in control and HERC2-depleted cells. **g**, **h** Cells were fixed and subjected to immunostaining analysis with anti-α-tubulin (green) and anti-CP110 (red) antibodies. Representative images of mitotic cells are shown. The frequencies of cells with various phenotypes were scored. **i**–**k** Control and *NudCL2* KO U2OS cells transfected with Flag-HERC2 or Flag vector for 48 h were subjected to western blot and immunofluorescence analyses using the indicated antibodies. β-actin, a loading control. The frequencies of cells with more than 4 centrin or 4 CP110 dots were plotted. **l–n** U2OS cells transfected with vectors and siRNAs for 72 h were subjected to western blot and immunofluorescence analyses using the indicated antibodies. β-actin, a loading control. The frequencies of cells with more than four centrin and four CP110 dots were plotted, respectively. DNA was visualized with 4′,6-diamidino-2-phenylindole (DAPI). Scale bars, 10 μm. Quantitative data are expressed as the mean ± SD (at least three independent experiments). More than 150 cells were counted in each experiment. *p < 0.05, **p < 0.01, ***p < 0.001, ns, not significanct, Student’s *t* test

HERC2 has been reported to participate in many fundamental cellular processes, including DNA repair, DNA replication, and PCM morphology maintenance^19^. Nevertheless, whether HERC2 plays a role in centriole duplication is still unknown. To examine the role of HERC2 in centriole duplication, we employed two small interfering RNAs (siRNAs) targeting different *HERC2* mRNA regions (siHERC2-1 and -2). Western blot analysis showed that the HERC2 proteins were efficiently downregulated at 72 h post transfection (Fig. 5a). Importantly, immunostaining with anti-centrin and anti-CP110 antibodies revealed an approximately three-fold increase in the number of cells with more than four centrioles in HERC2-depleted cells compared to the control (Fig. 5b–d). Flow cytometry data showed that knockdown of HERC2 had no significant effect on cell cycle progression (Fig. 5e, f). Furthermore, downregulation of HERC2 resulted in a significant increase in mitotic cells with pseudobipolar spindles (Fig. 5g, h). Thus, these results strongly suggest that HERC2 plays an important role in restraining centriole amplification.

Since our data show that NudCL2 interacts with and stabilizes HERC2 and that both NudCL2 downregulation and HERC2 depletion lead to centriole amplification (Figs. 2, 4, and 5), we next asked whether NudCL2 negatively regulates centriole duplication through HERC2. Exogenous expression of HERC2 efficiently reversed centriole amplification in *NudCL2* KO cells (Fig. 5i–k). By contrast, ectopic expression of NudCL2 failed to rescue the defects induced by HERC2 depletion (Fig. 5l–n). Collectively, these data suggest that HERC2 functions as downstream of NudCL2 to restrain centriole amplification.

### USP33 is involved in the HERC2-mediated regulation of centriole duplication in *NudCL2* KO cells

**Fig. 6:**
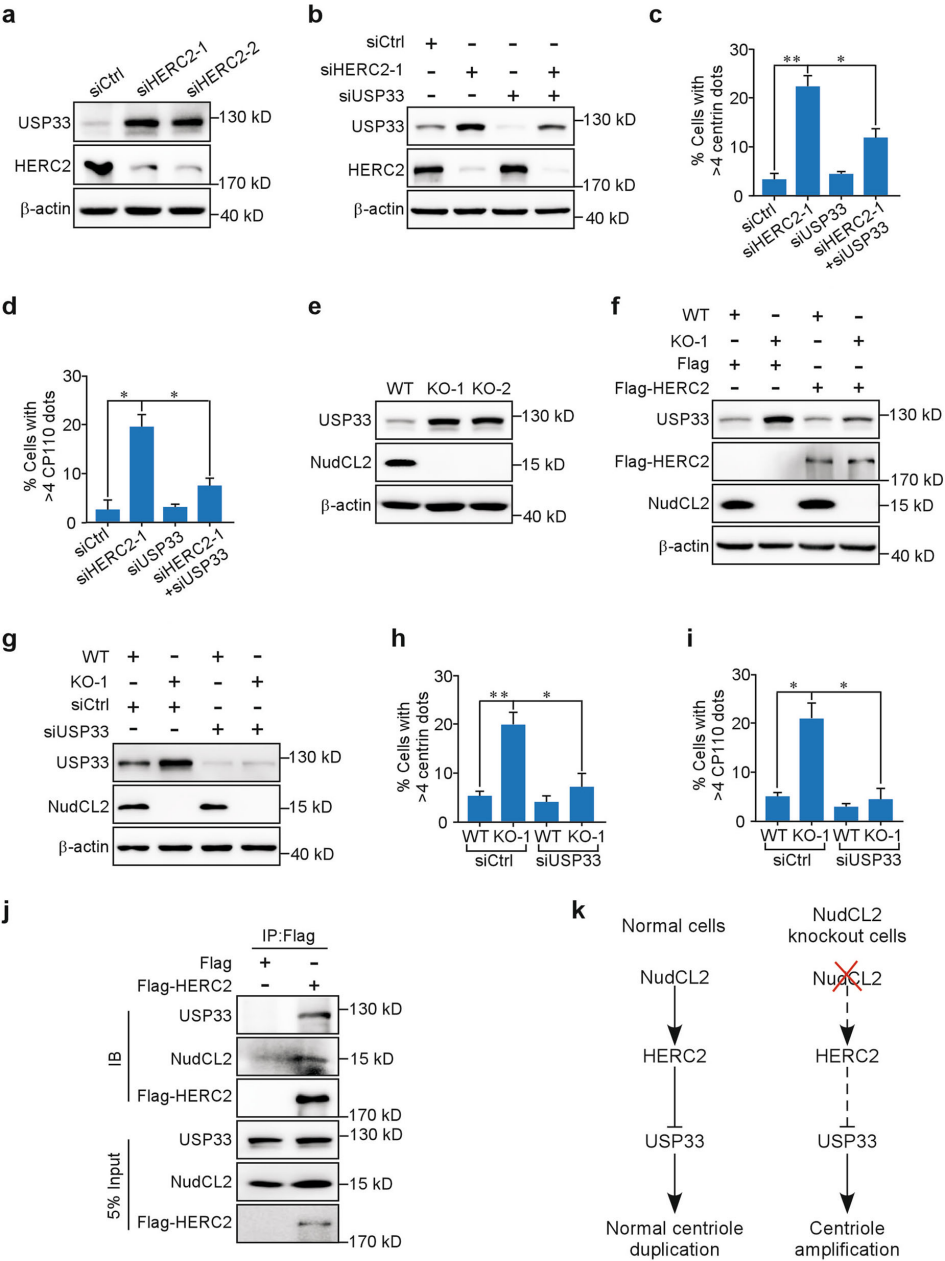
Ubiquitin-specific peptidase (USP33) is involved in HECT domain and RCC1-like domain-containing protein 2 (HERC2)-mediated centriole duplication in nuclear distribution gene C-like protein 2 (*NudCL2*) knockout (KO) U2OS cells. **a** Western blot analysis of USP33 protein levels in control and HERC2-depleted U2OS cells. β-actin, a loading control. **b–d** U2OS cells transfected with the indicated small interfering (siRNAs) for 48 h were processed for western blot and immunofluorescence analyses with the indicated antibodies. β-actin, a loading control. The frequencies of cells with more than four centrin and four CP110 dots were calculated, respectively. **e** Western blot analysis of USP33 protein levels in control and *NudCL2* KO U2OS cells. β-actin, a loading control. **f** Cells transfected with Flag-HERC2 or Flag vector for 48 h were subjected to western blot analysis with the indicated antibodies. β-actin, a loading control. **g–i** Control and *NudCL2* KO U2OS cells transfected with control or USP33 siRNAs for 48 h were subjected to western blot and immunofluorescence analyses with the indicated antibodies. β-actin, a loading control. The frequencies of cells with more than four centrin and four CP110 dots were plotted, respectively. **j** U2OS cells transfected with Flag-HERC2 or Flag vector for 48 h were harvested and subjected to immunoprecipitation analysis using the indicated antibodies. Quantitative data are expressed as the mean ± SD (at least three independent experiments). More than 300 cells were counted in each experiment. *p < 0.05, **p < 0.01, Student’s *t* test. **k** Working model for the role of NudCL2 in centriole duplication. NudCL2 restrains centriole amplification by stabilizing HERC2. Knockout of NudCL2 leads to HERC2 degradation and USP33 accumulation, resulting in centriole amplification

Given that the E3 ligase HERC2 target protein ubiquitin-specific peptidase 33 (USP33) plays an important role in the positive regulation of centriole duplication^20,21^, we asked whether USP33 acts as a downstream effector of the NudCL2/HERC2 axis to restrain centriole amplification during the cell cycle. First, we tested USP33 protein levels in HERC2-depleted cells and found that USP33 accumulated after HERC2 knockdown (Fig. 6a), consistent with a previous study^20^. Downregulation of USP33 reversed centriole amplification in HERC2-depleted cells (Fig. 6b–d), suggesting that HERC2 may control accurate centriole duplication through USP33. Next, we examined whether the protein stability of USP33 was regulated by NudCL2 and found that USP33 was obviously increased in *NudCL2* KO cells compared to the control (Fig. 6e). Further analyses showed that *NudCL2* KO decreased the degradation of USP33 (Supplementary Fig. 5). Importantly, ectopic expression of HERC2 reversed centriole amplification and USP33 accumulation induced by NudCL2 deletion (Figs. 5i–k, 6f). Knockdown of USP33 suppressed centriole amplification in *NudCL2* KO cells (Fig. 6g–i). Moreover, immunoprecipitation experiments showed that HERC2 was associated with NudCL2 and USP33 (Fig. 6j). Taken together, our results indicate that USP33 functions as a downstream effector of the NudCL2/HERC2 pathway to restrain centriole overduplication (Fig. 6k).

## Discussion

Centriole duplication is tightly controlled during cell cycle progression^1^. Accumulating studies have revealed that a number of proteins are involved in restraining centriole amplification, such as Krüppel-like factor 14, RNA-binding motif protein 14, cell division cycle 6, and so on^22–27^. However, the molecular mechanism of suppressing centriole amplification is still a mystery. In this report, we provide evidence that NudCL2 plays an essential role in restraining centriole amplification in mammalian cells. Downregulation of NudCL2 results in extranumerary centrioles (Fig. 2, Supplementary Figs. 1 and 2), and overexpression of NudCL2 inhibits HU-induced centriole overduplication (Fig. 3). NudCL2 is found to interact with and stabilize HERC2 (Fig. 4). Ectopic expression of HERC2 reverses centriole amplification induced by NudCL2 deletion (Fig. 5i–k). Either loss of NudCL2 or depletion of HERC2 increases USP33 protein levels (Fig. 6a, e, f). Moreover, downregulation of USP33 reverses centriole amplification induced by NudCL2 KO or HERC2 knockdown (Figs. 6b–d, g–i). Thus, these data indicate a hitherto undescribed mechanism in which NudCL2 functions as an important checkpoint protein to restrain centriole amplification by stabilizing HERC2 to decrease USP33 (Fig. 6k).

Emerging studies have identified a number of centrosomal proteins such as polo-like kinase 1 (Plk1), polo-like kinase 4 (Plk4), spindle assembly abnormal protein 6 (SAS6), separase, and SCL/TAL1-interrupting locus (STIL) that play crucial roles in the centriole duplication cycle^1^. During centriole biogenesis, Plk4 is recruited to the base of centrioles by centrosomal protein of 152 kDa and centrosomal protein of 192 kDa^28,29^. Then, Plk4 phosphorylates STIL and triggers the recruitment of SAS6 to centrioles, which initiates centriole assembly^30–32^. Additionally, Plk1 and separase participate in centriole disengagement, a critical licensing step for centriole duplication in the next cell cycle^33^. Overexpression of these proteins induces centriole amplification^34–38^. Here, we found that the NudCL2/HERC2/USP33 axis is essential for the precision regulation of centriole duplication. Intriguingly, our data reveal that NudCL2 deletion has no obvious effect on the protein levels of the above regulators, including Plk1, Plk4, SAS6, separase, and STIL (Supplementary Fig. 6), suggesting an unknown mechanism underlying the role of NudCL2 in centriole duplication.

Accumulating data implicate that HERC2 plays important roles in many cellular processes. HERC2 is required for the proper maturation of double-strand break responses by promoting the retention of DNA repair factors on damaged chromosomes^39^. HERC2 promotes DNA replication by facilitating minichromosome maintenance complex component 2 phosphorylation^40^. In addition, HERC2 has been found to participate in cell cycle regulation by inhibiting G2–M checkpoint activity via destabilizing breast cancer type 1 susceptibility protein^41^. Recently, HERC2 has been reported to localize to centrosomes and maintain PCM morphology by interacting with and ubiquitinating neuralized E3 ubiquitin protein ligase 4^42^; however, whether HERC2 plays a role in centriole duplication remains unknown. Here, our data show that downregulation of HERC2 leads to centriole amplification and pseudobipolar spindle formation in mammalian cells, suggesting a role of HERC2 in the accurate control of centriole duplication (Fig. 5). USP33 is a HERC2 target protein and participates in positively regulating centriole duplication^20,21^. In this study, we find that USP33 is accumulated in HERC2-depleted cells. Downregulation of USP33 is able to effectively reverse centriole amplification induced by HERC2 knockdown. These data suggest a previously uncharacterized function of HERC2 in restraining centriole amplification by destabilizing USP33.

In this study, we found that downregulation of NudCL2 causes HERC2 degradation (Fig. 4); however, the mechanism underlying the stabilization of HERC2 regulated by NudCL2 is still unknown. NudCL2 contains a core structure of p23 (p23 domain), which acts as an Hsp90 cochaperone to regulate the folding and maturation of client proteins^9^. Our recent work showed that NudCL2 functions as an Hsp90 cochaperone to stabilize cohesin subunits by modulating Hsp90 ATPase activity^10^. Based on these scenarios, further studies are clearly needed to determine whether Hsp90 is involved in the regulation of HERC2 stability.

Vertebrate NudC has three homologs: NudC, NudCL, and NudCL2, all of which have been reported to localize at centrosomes, an important organelle for cell cycle and ciliogenesis^6,43–45^. Depletion of NudC leads to multiple mitotic defects including the multipolar spindles and the lagging chromosomes, and causes ciliary defects in mammalian cells and zebrafish^46,47^. Downregulation of NudCL results in the similar phenotypes to that of NudC depletion during mitosis^48^. NudCL depletion also influences cilia assembly in interphase^49^. Here, our data indicate that NudCL2 acts as a centrosome duplication checkpoint protein to restrain centriole amplification by destabilizing HERC2. Taken together, these studies suggest that members of the NudC family function as important regulators of centrosome function.

## Materials and methods

### Plasmids, primers, and oligonucleotides

The *GFP-NudCL2* and *Myc-NudCL2* plasmids were constructed as described previously^6^. Plasmids expressing Flag-tagged full-length *HERC2* were obtained from Addgene (55613)^20^, and the sequence of *HERC2* was confirmed by DNA sequencing. All siRNAs were designed or synthesized by GenePharma (Shanghai, China). The sense sequences of the siRNA duplexes are as follows:

siHERC2-1: 5′-GGAAAGCACUGGAUUCGUUTT-3′^30^;

siHERC2-2: 5′-GAAGGUGGCUGUUCACUCATT-3′^30^;

siUSP33: 5′-GAUCAUGUGGCGAAGCAUATT-3′^21^;

siNudCL2-1: 5′-ACCUUGAGAAAUAACTGCUTT-3′^10^;

siNudCL2-2: 5′-GACUUCUCUACUAGAAUCUTT-3′.

### Cell culture, transfection, and drug treatment

U2OS, HeLa, and HEK-293 cells were maintained in Dulbecco’s modified Eagle’s medium (Corning, Shanghai, China) supplemented with 10% fetal bovine serum (PAA Laboratories, Northbrook, IL, USA). The human colorectal adenocarcinoma cell line DLD1 and the human breast cancer cell line CAL51 were cultured in RPMI-1640 (Corning) supplemented with 10% fetal bovine serum (PAA Laboratories). Cells were cultured in humidified incubators at 37 °C with 5% CO_2_. Transfections of plasmids and siRNAs were carried out using polyjet (SignaGen Laboratories, Rockville, MD, USA) and Lipofectamine RNAiMAX (Invitrogen, Carlsbad, CA, USA) according to the manufacturer’s instructions, respectively. In the centriole-overduplication assay, 4 mM HU (Sigma-Aldrich, St. Louis, MO, USA) was used to treat U2OS cells for 48 h. For CHX (Sigma-Aldrich, St. Louis, MO, USA) chase analysis, 100 µg/ml CHX was used for the indicated times as described in the text. To block the proteasome-dependent degradation pathway, 1 µM MG132 (Millipore, Billerica, MA, USA) was added to U2OS cells for 2 h.

### Generation of *NudCL2* KO cell lines by CRISPR/Cas9-mediated genome editing

The sgRNA, 5′-GAAGTTCAGGTGCCGCC-3′, targeting the first exon of the *NudCL2* gene was designed and synthesized by Nanjing YSY Biotech Ltd (Nanjing, China). Then, the CRISPR/Cas9 plasmid was constructed by cloning the sgRNA into its backbone. U2OS, HEK-293, and DLD1 cells were transfected with this plasmid for 48 h followed by treatment with 1 μg/ml puromycin for 48 h. After selection, the cells were counted and diluted to a density of 1 cell per 200 µl of medium and seeded into 96-well plates to obtain single colonies. *NudCL2* KO colonies were identified by western blot and genomic DNA sequencing analyses. The primers used to amplify the target region are as follows:

forward: 5′-AGGCGTAGCCTAAGCGTGGGATTC-3′;

reverse: 5′-ACCCAACAGTCGTTCAGGGAAACG-3′.

### Antibodies

An anti-NudCL2 antibody was generated as described previously^6^. Antibodies against centrin 1 (Millipore, Billerica, MA, USA), CP110 (Proteintech, Wuhan, China), γ-tubulin (Sigma-Aldrich, St. Louis, MO, USA), α-tubulin (Sigma-Aldrich, St. Louis, MO, USA), β-actin (Sigma-Aldrich, St. Louis, MO, USA), Plk1 (Sigma-Aldrich, St. Louis, MO, USA), Flag (Beyotime Biotechnology, Shanghai, China), c-Myc (Santa Cruz Biotechnology, CA, USA), SAS6 (Santa Cruz Biotechnology, CA, USA), separase (Santa Cruz Biotechnology, CA, USA), CDK2 (Santa Cruz Biotechnology, CA, USA), cyclin A (Santa Cruz Biotechnology, CA, USA), cyclin E (Santa Cruz Biotechnology, CA, USA), STIL (Abcam, Cambridge, MA, USA), HERC2 (BD Biosciences, San Jose, CA, USA; Bethyl Laboratories, Montgomery, TX, USA), Plk4 (Proteintech, Wuhan, China), and USP33 (Proteintech, Wuhan, China) were acquired commercially.

### Immunoprecipitation and western blot

Immunoprecipitation was performed as previously described^6,50^. Briefly, cells were lysed in TBSN buffer (20 mM Tris [pH 8.0], 150 mM NaCl, 0.5% Nonidet P-40, 5 mM EGTA, 1.5 mM EDTA, 0.5 mM Na_3_VO_4_, 20 mM *p*-nitrophenyl phosphate) containing a cocktail of protease inhibitors (Roche, Basel, Switzerland) and then subjected to immunoprecipitation with the indicated antibodies or anti-Flag antibody-coupled beads (Sigma-Aldrich, St. Louis, MO, USA). The proteins were separated in a sodium dodecyl sulfate (SDS)-polyacrylamide gel electrophoresis gel and transferred to a polyvinylidene fluoride membrane (Millipore, Billerica, MA, USA). The membranes were blocked with 5% skim milk at room temperature for 1 h, then incubated with the indicated primary antibodies and corresponding horse radish peroxidase-conjugated secondary antibodies (Cell Signaling Technology, Beverly, MA, USA), and, finally, detected by enhanced chemiluminescence (Fude biological technology, Hangzhou, China) according to the manufacturer’s instructions.

### Immunofluorescence

Cells grown on coverslips were fixed with cold methanol at −20 °C for 5 min, permeabilized in 0.1% Triton X-100/phosphate-buffered saline (PBS) for 15 min, blocked with 3% bovine serum albumin/PBS for 30 min, and then incubated with the primary antibodies indicated in the text for 2 h at room temperature. After washing with 0.1% Triton X-100/PBS for 15 min, cells were incubated with secondary antibodies (Alexa Fluor 488- or 568-conjugated anti-rabbit or mouse IgG, Invitrogen) for 1 h at room temperature. DNA was stained with 4′,6-diamidino-2-phenylindole (DAPI) (Beyotime Technology, Shanghai, China). Finally, the mounted coverslips were analyzed by confocal fluorescence microscopy (IX81-FV1000, Olympus, Japan).

### Fluorescence-activated cell sorting

For cell cycle analysis, cells were collected and washed with PBS and fixed in cold 70% ethanol. The samples were washed twice with PBS and incubated with a staining solution containing 20 µg/ml propidium iodide (Beyotime Technology, Shanghai, China) and 20 μg/ml RNase A (Beyotime Technology, Shanghai, China) at 37 °C for 30 min. The samples were analyzed using a flow cytometer (Cytomic FC 500 MCL, Beckman Coulter, Brea, CA, USA).

### Quantitative RT-PCR

Total RNA was extracted with Trizol (Invitrogen) and reverse transcribed to obtain complementary DNA with HiScript Q RT SuperMix (Vazyme, Nanjing, China). Quantitative RT-PCR analysis for *HERC2* mRNA was performed using a Bio-Rad CFX-Totch System (Bio-Rad Laboratories, Hercules, CA, USA). Primers used to amplify the target region are as follows:

forward: 5′-TGAAGAAGAAACTCCTGCACCT-3′;

reverse: 5′-GGTGGTGGCTGACTGGAC-3′.

### Centrosome isolation

Centrosome purification was performed as previously described with some modifications^51^. U2OS cells that had been treated with a solution containing 10 μg/ml nocodazole (Santa Cruz Biotechnology, CA, USA) and 5 μg/ml cytochalasin B (Sigma-Aldrich, St. Louis, MO, USA) for 1.5 h were lysed with lysis buffer (1 mM HEPES [pH 7.2], 0.5% NP-40, 0.5 mM MgC1_2_, 0.1% β-mercaptoethanol and protease inhibitors) on ice for 20 min. Swollen nuclei and chromatin aggregates were removed by centrifugation (2500 × *g*, 10 min). The supernatant was supplemented with HEPES buffer to a final concentration of 10 mM and incubated with 2 Units/ml of DNase I (Sigma-Aldrich, St. Louis, MO, USA) on ice for 30 min. The lysate was underlaid with 1 ml of 60% sucrose solution and centrifuged at 25,000 × *g* for 30 min at 4 °C. The crude centrosome preparation was diluted with lysis buffer and layered onto a discontinuous sucrose gradient (from bottom to top, containing 0.5, 0.3, and 0.3 ml of 70%, 50%, and 40% sucrose solutions, respectively) in a 5 ml tube, followed by centrifugation at 97,000 × *g* for 1.5 h at 4 °C. Subsequently, fractions were collected, diluted with 10 mM PIPES (pH 7.2), and centrifuged at 14,000 rpm for 10 min to pellet the centrosomes. The centrosome pellets were resuspended in 40 µl of SDS sample buffer.

### Statistical analysis

All experiments were repeated at least three times. Two-tailed Student’s *t* tests were used for comparisons between two groups (GraphPad Prism 5).

## Supplementary information

Supplementary figure legends

Loss of NudCL2 causes centriole amplification in DLD1 cells

Knockdown of NudCL2 leads to centriole amplification in CAL51 cells

Depletion or loss of NudCL2 causes HERC2 downregulation

Deletion of NudCL2 increases the degradation of HERC2 in U2OS cells

Loss of NudCL2 decreases the degradation of USP33 in U2OS cells

Knockout of NudCL2 has no obvious effect on the protein levels of the crucial centriole duplication regulators
