# Supplementary material for: NudC-like protein 2 restrains centriole amplification by stabilizing HERC2
Source: Cell Death Dis. 2019 Aug 19;10(9):628. doi: 10.1038/s41419-019-1843-3 (PMC6700069; doi:10.1038/s41419-019-1843-3)
Supplement: Supplementary file 1 — Supplementary figure legends [file 41419_2019_1843_MOESM1_ESM.docx]

**Supplementary Figure Legends**

**Supplementary Fig. 1 Loss of NudCL2 causes centriole amplification in DLD1 cells. a** Deletion mutation of the *NudCL2* DNA locus in *NudCL2* knockout DLD1 cells. **b** Western blot analysis of NudCL2 expression in control and *NudCL2* KO DLD1 cells. β-actin, a loading control. **c-e** Control and *NudCL2* KO DLD1 cells were fixed and processed for immunoﬂuorescence analysis with anti-centrin (green) and anti-CP110 (red) antibodies. Higher magnifications of the boxed regions are displayed. The frequencies of cells with more than 4 centrin and 4 CP110 dots were calculated, respectively. **f**, **g** Control and *NudCL2* KO DLD1 cells were fixed and stained with anti-γ-tubulin (green) and anti-CP110 (red) antibodies. Higher magnifications of the boxed regions are shown. The number of cells with more than 2 γ-tubulin dots was plotted. DNA was visualized with DAPI (blue). Scale bars, 10 μm. Quantitative data are expressed as the mean ± SD (at least three independent experiments). More than 300 cells were counted in each experiment. ***p* < 0.01, ****p* < 0.001, Student’s *t* test.

**Supplementary Fig. 2 Knockdown of NudCL2 leads to centriole amplification in CAL51 cells**. CAL51 cells were transfected with control or NudCL2 siRNAs for 72 h and subjected to the following analyses. **a** Western blot analysis of the expression of NudCL2. β-actin, a loading control. **b-d** Immunofluorescence analysis with anti-centrin (green) and anti-CP110 (red) antibodies was carried out to detect centriole amplification. Higher magnifications of the boxed regions are displayed. The frequencies of cells with more than 4 centrin and 4 CP110 dots were calculated, respectively. **e, f** Immunofluorescence analysis with anti-γ-tubulin (green) and anti-CP110 (red) antibodies was performed to detect centrosome amplification. Higher magnifications of the boxed regions are shown. The number of cells with more than 2 γ-tubulin dots was plotted. DNA was visualized with DAPI (blue). Scale bars, 10 μm. Quantitative data are expressed as the mean ± SD (at least three independent experiments). More than 300 cells were counted in each experiment. **p* < 0.05, ***p* < 0.01, Student’s *t* test.

**Supplementary Fig. 3** **Depletion or loss of NudCL2 causes HERC2 downregulation. a** CAL51 cells transfected with control or NudCL2 siRNAs for 72 h were harvested and lysed. Lysates of the cells were subjected to western blot analysis using anti-HERC2 and anti-NudCL2 antibodies. β-actin, a loading control. **b** Control and *NudCL2* KO DLD1 cells were harvested and lysed. The cell lysates were subjected to western blot analysis using anti-HERC2 and anti-NudCL2 antibodies. β-actin, a loading control.

**Supplementary Fig. 4** **Deletion of NudCL2 increases the degradation of HERC2 in U2OS cells.** **a** Control and *NudCL2* KO U2OS cells treated with 100 μg/ml cycloheximide were harvested at different time points. The cell lysates were subjected to western blot analysis using anti-HERC2 and anti-NudCL2 antibodies. β-actin, a loading control. **b** ImageJ software was used to quantify the protein levels. The relative amounts of HERC2 were calculated after normalization (HERC2/β-actin).

**Supplementary Fig. 5** **Loss of** **NudCL2 decreases the degradation of USP33 in U2OS cells.** **a** Control and *NudCL2* KO U2OS cells treated with 100 μg/ml cycloheximide were harvested at different time points. Lysates of the cell were prepared and subjected to western blot analysis using anti-USP33 and anti-NudCL2 antibodies. β-actin, a loading control. **b** ImageJ software was used to quantify the protein levels. The relative amounts of USP33 were measured after normalization (USP33/β-actin).

**Supplementary Fig. 6 Knockout of NudCL2 has no obvious effect on the protein levels of the crucial centriole duplication regulators.** Control and *NudCL2* KO U2OS cells were harvested and lysed. The cell lysates were subjected to western blot analysis using the indicated antibodies. β-actin, a loading control.
