# Supplementary figures and images for: NudC-like protein 2 restrains centriole amplification by stabilizing HERC2
Source: Cell Death Dis. 2019 Aug 19;10(9):628. doi: 10.1038/s41419-019-1843-3 (PMC6700069; doi:10.1038/s41419-019-1843-3)

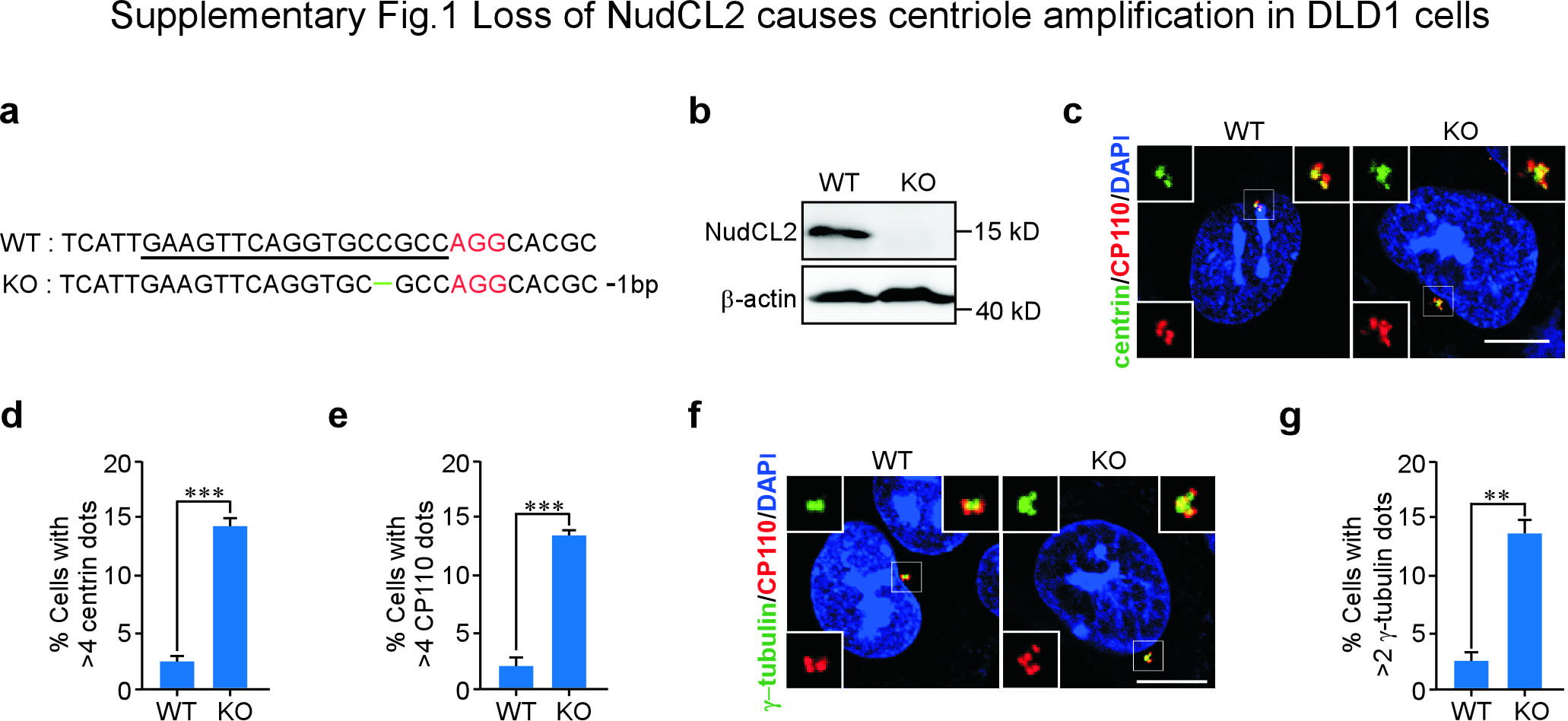

Supplement: Supplementary file 2 — Loss of NudCL2 causes centriole amplification in DLD1 cells [file 41419_2019_1843_MOESM2_ESM.jpg]

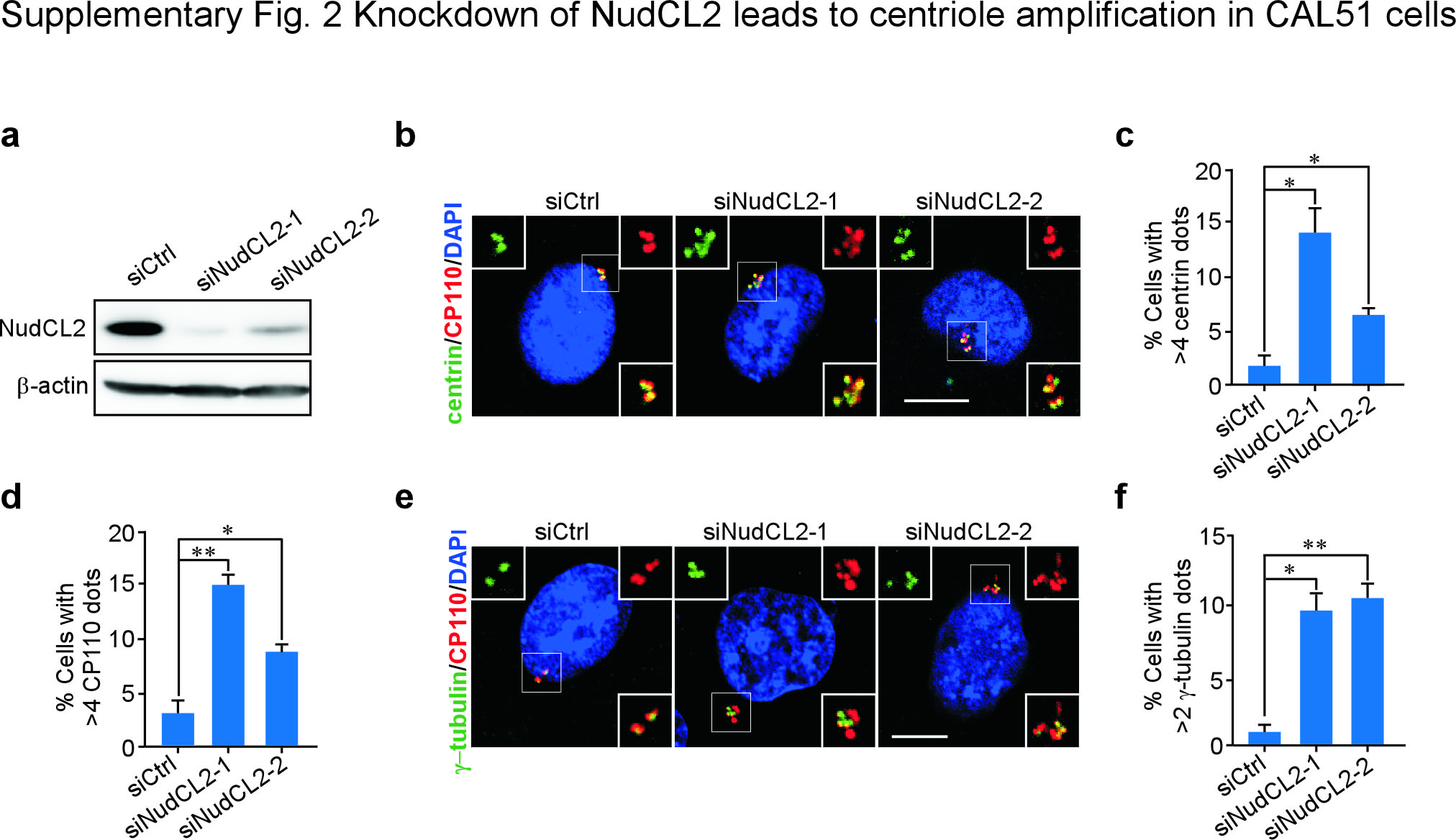

Supplement: Supplementary file 3 — Knockdown of NudCL2 leads to centriole amplification in CAL51 cells [file 41419_2019_1843_MOESM3_ESM.jpg]

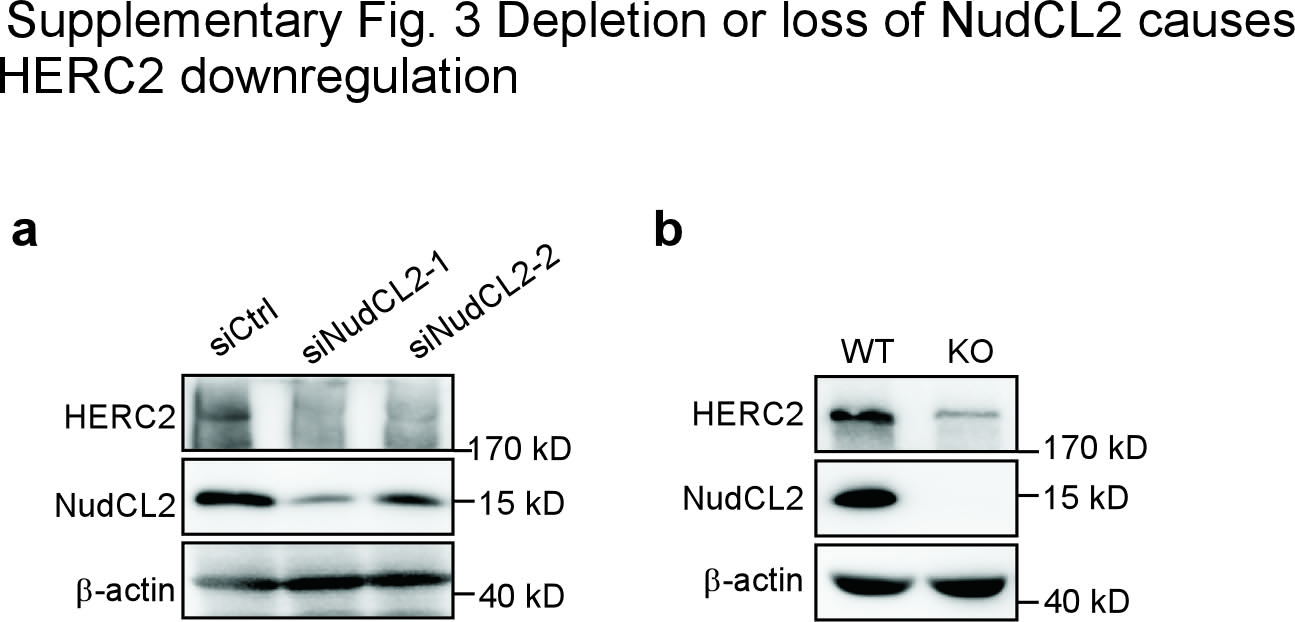

Supplement: Supplementary file 4 — Depletion or loss of NudCL2 causes HERC2 downregulation [file 41419_2019_1843_MOESM4_ESM.jpg]

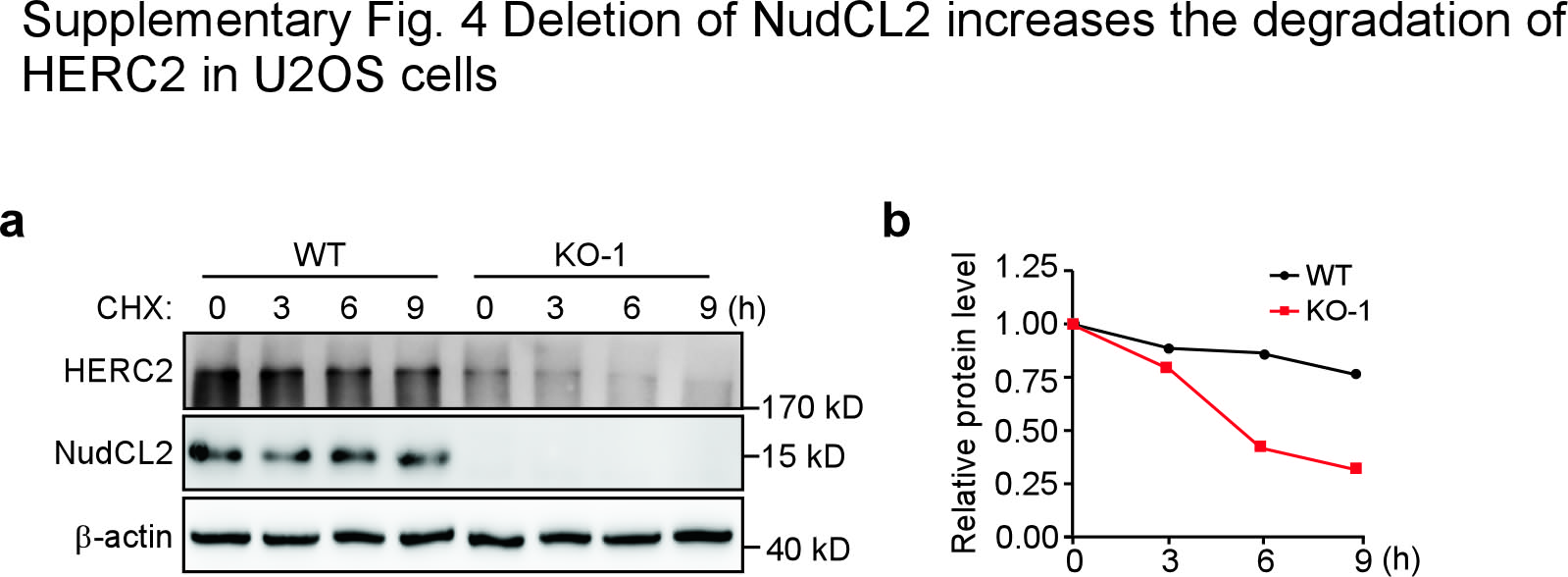

Supplement: Supplementary file 5 — Deletion of NudCL2 increases the degradation of HERC2 in U2OS cells [file 41419_2019_1843_MOESM5_ESM.jpg]

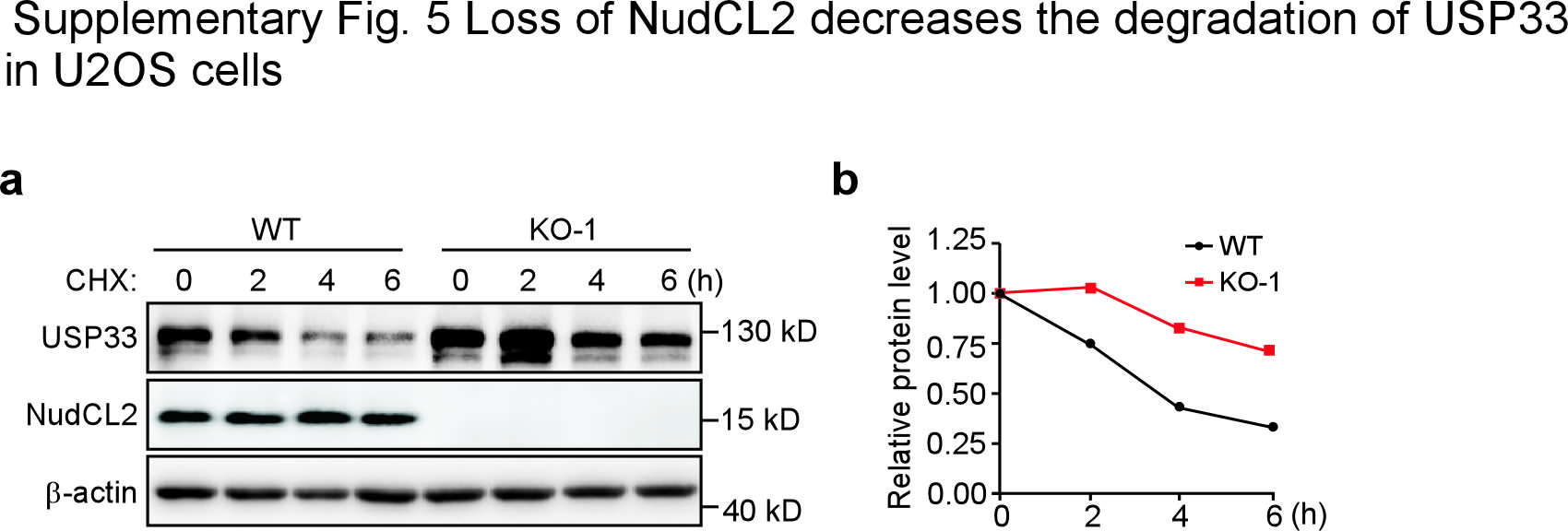

Supplement: Supplementary file 6 — Loss of NudCL2 decreases the degradation of USP33 in U2OS cells [file 41419_2019_1843_MOESM6_ESM.jpg]

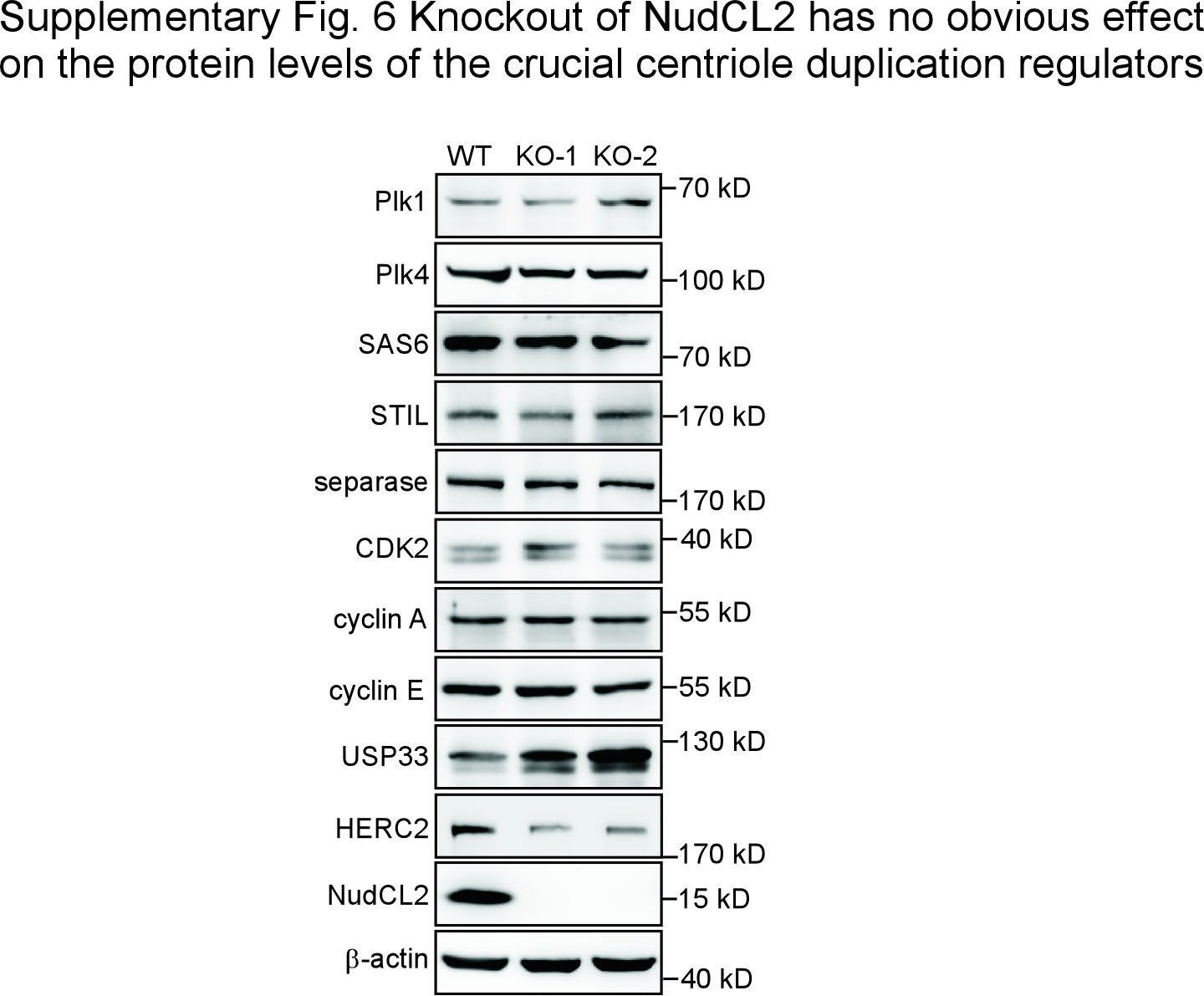

Supplement: Supplementary file 7 — Knockout of NudCL2 has no obvious effect on the protein levels of the crucial centriole duplication regulators [file 41419_2019_1843_MOESM7_ESM.jpg]
